# 

**Published:** 1879-08

**Authors:** 


No. 308. AUGUST, 1879. Price 15 Cts.

TWENTY-SIXTH YEAR.

### HALL'S

### Journal of Health.

E. H. GIBBS, A.M., M.D., Editor,

No. 141 EIGHTH STREET (near Broadway) NEW YORK.

**Terms, $1.50 a Year, or $1.00 for Eight Months. Postage paid.**

Couch & Johnston, Printers, 11 Frankfort Street, New York.

